# An Empirical Study of the Transmission Power Setting for Bluetooth-Based Indoor Localization Mechanisms

**DOI:** 10.3390/s17061318

**Published:** 2017-06-07

**Authors:** Manuel Castillo-Cara, Jesús Lovón-Melgarejo, Gusseppe Bravo-Rocca, Luis Orozco-Barbosa, Ismael García-Varea

**Affiliations:** 1 Computer Science School, Sciences Faculty, Center of Information and Communication Technologies, Universidad Nacional de Ingeniería, Lima 25, Peru; jlovonm@uni.pe (J.L.-M.); gbravor@uni.pe (G.B.-R.); 2 Albacete Research Institute of Informatics, Universidad de Castilla-La Mancha, 02071 Albacete, Spain; luis.orozco@uclm.es (L.O.-B.); ismael.garcia@uclm.es (I.G.-V.)

**Keywords:** indoor positioning, location fingerprinting, bluetooth, BLE4.0, supervised learning algorithm, signal processing, RSSI, multipath fading, transmission power

## Abstract

Nowadays, there is a great interest in developing accurate wireless indoor localization mechanisms enabling the implementation of many consumer-oriented services. Among the many proposals, wireless indoor localization mechanisms based on the Received Signal Strength Indication (RSSI) are being widely explored. Most studies have focused on the evaluation of the capabilities of different mobile device brands and wireless network technologies. Furthermore, different parameters and algorithms have been proposed as a means of improving the accuracy of wireless-based localization mechanisms. In this paper, we focus on the tuning of the RSSI fingerprint to be used in the implementation of a Bluetooth Low Energy 4.0 (BLE4.0) Bluetooth localization mechanism. Following a holistic approach, we start by assessing the capabilities of two Bluetooth sensor/receiver devices. We then evaluate the relevance of the RSSI fingerprint reported by each BLE4.0 beacon operating at various transmission power levels using feature selection techniques. Based on our findings, we use two classification algorithms in order to improve the setting of the transmission power levels of each of the BLE4.0 beacons. Our main findings show that our proposal can greatly improve the localization accuracy by setting a custom transmission power level for each BLE4.0 beacon.

## 1. Introduction

Nowadays, there is great interest in developing indoor localization algorithms making use of the latest developments on low-power wireless technologies. Among the latest developments, Bluetooth technologies are attracting the attention of many researchers. Their wide availability, practically all smartphones incorporate a Bluetooth interface, is behind the increasing interest in developing indoor localization-based services.

Most recent Bluetooth indoor localization systems are based on the Received Signal Strength Indication (RSSI) metric [1,2]. Recent studies have shown that Bluetooth Low Energy 4.0 (BLE4.0) signals are very susceptible [3] to fast fading impairments. This fact makes it difficult to apply the RSSI-distance models commonly used in the development of Wi-Fi-based localization mechanisms [4,5]. Recent studies proposed alternative methods, as, for example, the use of Voronoi diagrams [6] or the use of a probability distribution to match the best solution to the localization problem [7]. In the context of BLE4.0 beacons, in [8], a proposal based on the use of an Isomap and a Weighted k-Nearest Neighbor (WKNN) is presented. As in previous related works [9,10], we explore the use of two supervised learning algorithms: the k-Nearest Neighbour (k-NN) and the Support Vector Machine (SVM) algorithms [11]. We go a step further by exploring the benefits of individually setting the transmission power as a means to improve the quality of the RSSI fingerprint to be used by the learning algorithms.

**Figure 1:**
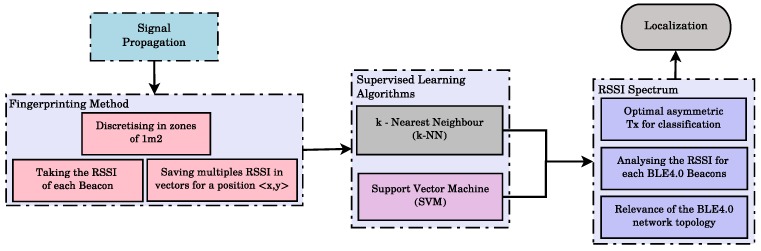
Overall schema proposal.

Figure 1 shows the overall proposed methodology. First, we analyse the capabilities of two mobile devices: a smartphone and a Raspberry Pi with a BLE4.0 antenna. Once the best device (in terms of the accuracy performance) has been selected, we study the relevance of every BLE4.0 beacon in our experimental environment. From this analysis, we conclude that an ad hoc setting of the transmission power level of the BLE4.0 beacons plays a major role on the quality of the signal fingerprint. In order to get a better insight on our findings, we pay particular attention on describing the floor plan of the lab premises. In fact, recent results show that the use of the floor plan as a basis to identify the multipath components may be exploited to enhance the accuracy of wireless indoor localization scheme [12]. Although the use of such schemes are still at their infancy and limited to wideband communications, they have revealed some insight on the impact of the structural features over the RSSI metric. In [12], Leit et al. have conducted several trials making use of ultra-wide band communications transceivers. Our main aim regarding this latter issue is to provide some insight on the impact of architectural features over the transmission power setting of the BLE4.0 beacons. To the best of our knowledge, this is the first study proposing an asymmetric transmission power setting of the BLE4.0 beacons. We then make use of two supervised learning algorithms to characterize the BLE4.0 beacon signal propagation. These algorithms will then be used for developing indoor localization mechanisms. The results obtained in a real-world scenario validate the proposal.

In the following, the paper is organized as follows. Section 2 reviews the related work and describes the main contribution of our work. Section 3 describes the experimental set-up including the challenges we can face when developing a BLE4.0 fingerprint-based localization mechanism. We also include a brief description of the two classification algorithms used on our proposal. In Section 4, we examine the adequacy of the experimental set-up on developing the localization scheme. Two main parameters are studied: (i) the contribution of each BLE4.0 beacon deployed in the environment; and (ii) the transmission power level of each BLE4.0 beacon. From this preliminary analysis, we conclude, in Section 5, that the accuracy of the localization mechanism can be improved by setting the transmission power of each BLE4.0 beacon at an appropriate level.

## 2. Related Work

Nowadays, the design of robust wireless indoor localization mechanisms is a very active research area. Among the many technologies available in the market nowadays, BLE4.0 beacons have spurred the interest of many practitioners and researchers. The main benefits of the technology rely on the installation and maintenance cost of the battery-operated BLE4.0 beacons. The development of a BLE-based indoor localization make use of the RSSI reported by the mobile devices—then, followed by one of two main approaches: Triangulation [13] and fingerprinting [14,15,16]. Lately, other approaches, such as context [17] and crowdsensing [18], are also being actively explored. Despite the efforts being carried out by the research community, the robust development of wireless indoor localization mechanism remains a major challenge. In this work, we are interested on improving the information obtained from the fingerprint of each individual BLE4.0 beacon. Since our goal is to develop the localization scheme based on a classification algorithm, we explore the benefits of setting the transmission power setting of each individual BLE4.0 beacon to improve the quality of the radio map (fingerprint). As in previous related works [10,15], we explore the use of two supervised learning algorithms: The k-Nearest Neighbour (k-NN) and the Support Vector Machine (SVM) algorithms [11]. In the sequel, we briefly review the most relevant works recently reported in the literature and point out the main aim of our work.

In [14], Kriz et al. have developed a localization comprising a set of Wi-Fi Access Points (AP) supplemented by BLE4.0 devices. The localization mechanism was based on the Weighted-Nearest Neighbours in Signal Space algorithm. Two of the main goals of this study have been to enhance the accuracy of wireless indoor localization by introducing the use of the BLE4.0 devices and the deployment of an information system being continuously updated by the RSSI levels reported by the mobile devices. Two main system parameters related to the BLE4.0 devices were varied to verify the performance of the indoor localization mechanism, namely, the scanning duration and the BLE4.0 beacons density. However, the transmission power was set to its maximum value all along the experimental trials.

In [15], the authors conduct an experimental study using 19 BLE4.0 beacons. Their study includes an analysis of the transmission power used by the BLE4.0 beacons over the accuracy of a BLE-based indoor localization scheme. Their results show that their initial power setting, set at the highest available level, was unnecessarily high for their deployment and that an attenuation of up to 25 dB would have had a low impact on the positioning accuracy. Different to our main aim, they were interested in identifying the attenuation bounds ensuring 100% availability of positioning, while avoiding a configuration consisting of proximity “islands”. All along their experimental fields trials, all BLE4.0 beacons were configured using the same transmission power setting. Their results also provide some insights on the tradeoffs between the number of BLE4.0 beacons required and the transmission power settings.

In [16], Paek et al. evaluate the accuracy in proximity and distance estimation of three different Bluetooth devices. Towards this end, they explore the setting of various transmission power levels. Besides finding that the three device brands vary substantially in the transmission power configuration, they conclude that the best power setting will depend on the actual aim of the localization mechanism. They conclude that higher transmission power will better fit to cover larger areas, while low transmission power should be used to detect the proximity of the target to a given area (BLE4.0 beacon). They conclude that the accuracy and efficiency of location estimation heavily depend on the accuracy of the measured RSSI measurements and the model used to estimate the distance and other environmental characteristics. In fact, one of their main claims is the need of a novel approach to overcome some of the main challenges faced by RSSI dynamics. In this work, we first examine the RSSI dynamics using two different devices: A commercial Android smartphone and a Raspberry Pi equipped with a BLE4.0 antenna. From a preliminary analysis, and one having identified the benefits of using the BLE4.0 antenna, we introduce a novel approach based on the use of an asymmetric transmission power setting of the BLE4.0 beacons. Our main aim to improve the quality of the information to be used to feed the classification algorithms. To the authors knowledge, the use of an asymmetric transmission power setting has not been explored on improving the accuracy of a BLE-based indoor localization algorithm.

## 3. BLE4.0 Indoor Localization: Set-Up, Tools and Algorithms

In this section, we introduce the specifications and technical details of our experimental setting. First, we describe the physical layout of the testbed that we have used to carry all indoor localization experiments. Next, the capabilities of two different mobile devices are experimentally assessed. Finally, the two classification algorithms used in our experiments are described.

### 3.1. Experimental Indoor Set-Up

**Figure 2:**
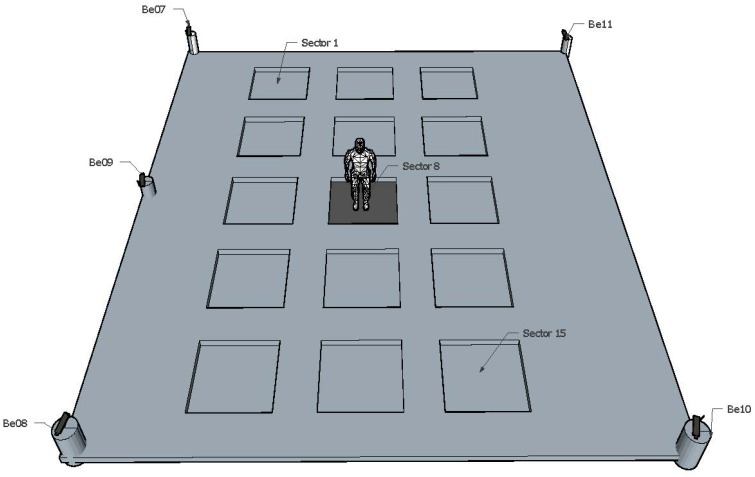
BLE4.0 beacon indoor localization set-up.

**Figure 3:**
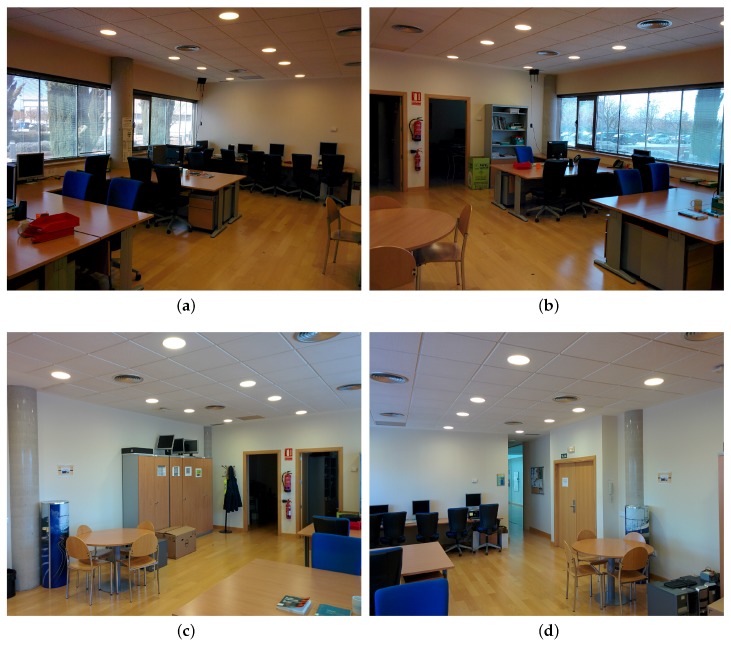
Pictures from each one of the four corners of the lab. (**a**) from Be07; (**b**) from Be08; (**c**) from Be10; (**d**) from Be11.

Our experiments were conducted in a lab of our research institute. We placed four BLE4.0 beacons at each one of the four corners of a 9.3 m by 6.3 m rectangular area. A fifth BLE4.0 beacon was placed in the middle of one of the longest edges of the room. Figure 2 depicts the experimental area where the five BLE4.0 beacons have been labelled as ’Be07’, ’Be08’, ’Be09’, ’Be10’ and ’Be11’. We divided the experimental area into 15 sectors of 1 m${}^{2}$ each separated by a guard sector of 0.5 m${}^{2}$. A 1.15 m-wide strip was left around the experimental area. This arrangement will allow us to better differentiate the RSSI level of joint sectors when reporting our results. Measurements were taken by placing the mobile device at the centre of each one of the 15 sectors as described below. The shortest distance between a BLE4.0 beacon and the receiver was limited to 1.5 m. Figure 3 shows four views taken from each one of the four corners of the lab. As seen from the figure, we have placed BLE4.0 beacons ’Be10’ and ’Be11’ in front of a window, Figure 3a,b, while all of the other BLE4.0 beacons have been placed in front of the opposite plasterboard wall. We further notice that BLE4.0 beacon ’Be08’ has been placed by the left edge of the entrance door, close to the a corridor with a glass wall, Figure 3d. Our choice has been based on recent results reported in the literature claiming that knowing the geometry of the experimental environment space may be exploited to develop more accurate indoor localization mechanisms [12].

According to the specifications of the five BLE4.0 beacons used in our experiments, they may operate at one of eight different transmission power (Tx) levels. Following the specifications, the transmission power levels are labelled in consecutive order from the highest to the lowest level as Tx=0x01,Tx=0x02,⋯,Tx=0x08 [19]. During our experiments, we conducted various measurement campaigns by fixing the transmission power level of all of the BLE4.0 beacons at the beginning of each campaign. Furthermore, all measurements were taken under line-of-sight conditions.

### 3.2. Bluetooth Receiver’s Characteristics

Receiver devices are very sensitive when used in indoor localization [20]. We start by assessing the capabilities of the two mobile devices: a smartphone running the Android 5.1 operating system, and a Raspberry Pi 2 equipped with a USB BLE4.0 antenna [21], from now on referred to as the smartphone and the BLE4.0 antenna, respectively. Furthermore, we will refer to each one of the 151 m${}^{2}$ sectors by a number from 1 to 15, where the sectors are numbered from left to right starting from the upper left corner.

We carried out a survey campaign as follows:

- We fixed the transmission power of all BLE4.0 beacons to the same level.
- We placed the mobile device at the centre of each one of the 151 m${}^{2}$ and measured the RSSI of each one of the five BLE4.0 beacons for a time period of one minute.
- We evaluated the mean and standard deviation of the RSSI for each one of the five BLE4.0 beacons.

The survey was carried out through a time period of five days evenly distributed between the morning and evening hours. The lab occupancy was limited to six people: Two of them were in charge of collecting the data, two other scientists working at the room located at one end of the lab, and the other two scientists at a different area connected with our scenario by means of a corridor. Sporadically, these people passed through the lab during the measurement campaign. This survey campaign was repeated three times in a time span of one month in order to provide different real life conditions and variability to the data gathering process.

**Figure 4:**
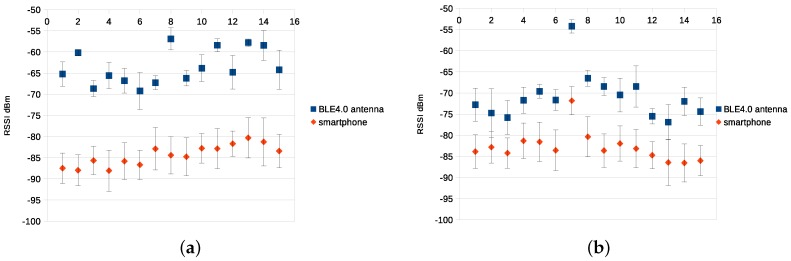
RSSI (dBm) for BLE4.0 Antenna and smartphone with transmission power Tx=0x04 for each sector (1.15) of our environment. (**a**) for Be07; (**b**) for Be09.

It is worth mentioning that the sampling rate of the smartphone is limited to 15 samples/second, while we have set a sampling rate of the BLE4.0 antenna to 86.6 samples/second. In fact, we were unable to match the sampling rates of both devices. Figure 4a,b show the average and standard deviation of the RSSI values for BLE4.0 beacons ’Be07’ and ’Be09’, respectively, using Tx=0x04. Since the purpose of this first experiment was to evaluate the capabilities of both mobile devices, the use of mid-power seemed to be the best choice. The figures show that the BLE4.0 antenna offers better results than the smartphone, higher RSSI levels and lower standard deviation.

### 3.3. Bluetooth Signal Attenuation

In the previous section, we have found that the first moment and standard deviation of the RSSI does not provide us with the means to determine the distance of a target device from a reference beacon. In this section, we further analyse the challenges faced when developing a localization scheme using as the main source of information the BLE4.0 RSSI levels. This analysis will allow us to motivate the use of supervised learning algorithm as a basis to develop wireless indoor localization mechanisms.

We focus now on the analysis of the traces of the RSSI data collected for BLE4.0 beacon ’Be07’ and ’Be10’. Our choice has been based on the fact that BLE4.0 beacon ’Be07’ and ’Be10’ have been placed at the two opposite corners of the lab. As seen in Figure 3c, BLE4.0 beacon ’Be07’ was placed close to the entrance of two office spaces, while ’Be10’ was placed by the window (see Figure 3a).

In the following, we analyse two different snapshots of the three data captures, denoted, from now on, as Take 1 and Take 2. The traces correspond to the data collected at sectors 4, 8 and 15. Be aware that, since we just counted with a BLE4.0 antenna, all traces were taken at different times of the day and at different dates. For simplicity, we will refer to Take 1 to the traces corresponding to the first data capture campaign; and by Take 2 to the traces resulting from the second data gathering campaign.

### Case 1: Sector 8

**Figure 5:**
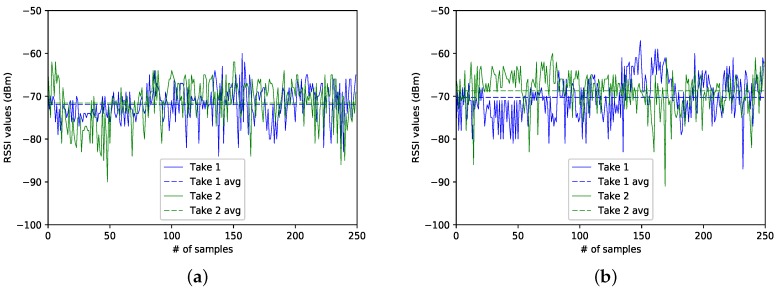
Sector 8: Comparison of the RSSI from different BLE4.0 beacons for Tx=0x04. (**a**) for Be07; (**b**) for Be10.

We start our analysis by examining the RSSI traces taken at Sector 8, the one corresponding to the sector located at the centre of the experimental area. Figure 5a,b show the two RSSI traces for each one of the two BLE4.0 beacons. We notice that, for a given BLE4.0 beacon, both traces show similar RSSI mean values (dashed lines). Since both BLE4.0 beacons were located at the same distance from the centre of the experimental area, we may expect to get similar average RSSI values for both BLE4.0 beacons. However, as seen from the figure, the RSSI average reported for BLE4.0 beacon ’Be10’ is higher than the one reported for BLE4.0 beacon ’Be07’. The main reason for this discrepancy may be explained by the fact that the BLE4.0 signals are highly sensitive to fast fading impairment: an issue that we will address in the following sections. This result is highly relevant since it clearly shows that we were quite successful in replicating our experiments: a must to set up a baseline scenario aiming to explore the impact of a given parameter over the performance of our proposal. It is also an important source of information to be exploited by the classification process.

### Case 2: Sector 4

**Figure 6:**
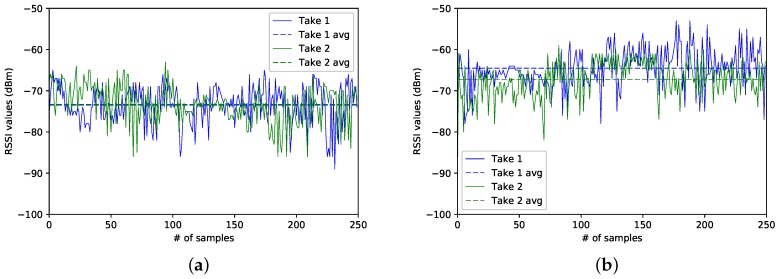
Sector 4: Comparison of the RSSI from different BLE4.0 beacons for Tx=0x04. (**a**) for Be07; (**b**) for Be10.

Figure 6a,b show the traces for both BLE4.0 beacons at Sector 4. In this case, BLE4.0 beacon ’Be07’ is closer to this sector than BLE4.0 beacon ’Be10’. However, as seen in the figures, the RSSI traces for BLE4.0 beacon ’Be07’ exhibit lower values than those reported for BLE4.0 beacon ’Be10’. It is also important to mention that, despite the captures for both beacons having been taken at different times, the average RSSI signal levels (dashed lines) of BLE4.0 beacon ’Be07’ for both traces were lower than the ones reported for the traces for BLE4.0 beacon ’Be10’. However, a more in-depth analysis of the impact of external actors over the signal should be conducted. For instance, a more in-depth study of the impact of the room occupancy and more importantly on how to integrate this parameter into the information to be fed to the classification algorithms should be studied.

### Case 3: Sector 15

**Figure 7:**
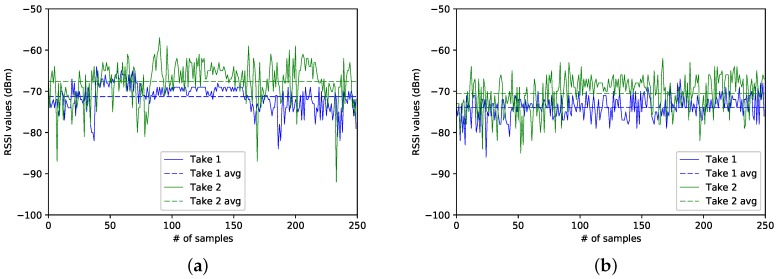
Sector 15: Comparison of the RSSI from different BLE4.0 beacons for Tx=0x04. (**a**) for Be07; (**b**) for Be10.

In this case, we analyse the traces collected at Sector 15, the closest sector to BLE4.0 beacon ’Be10’. As can be seen in Figure 7a,b, it is surprising that the average signal level (dashed lines) of BLE4.0 beacon ’Be07’ is higher than the average of the BLE4.0 beacon ’Be10’. This confirms once again that the signal is highly sensitive to the fast fading impairment. We also notice that the traces Take 1 for both BLE4.0 beacons are smoother than the traces obtained during the second campaign, Take 2. The high variance of Take 1 of BLE4.0 beacon ’Be07’ can be explained by the fact that the way from the main door of the lab into the offices passes between the location of BLE4.0 beacon ’Be07’ and Sector 15. This shows the importance of counting with an estimate of the room occupancy as a key parameter to develop accurate wireless indoor localization mechanisms. It also shows the benefits of counting with a baseline scenario to guide the classification task and identify the relevance of other key parameters. In our case, we are interested here in exploring the proper setting of the transmission power of the BLE4.0 beacons.

The above analysis of the statistics of the data collected reveal that Bluetooth signals are very susceptible to fast fading impairments. They also show, up to a certain extent, the impact of the occupancy over the signal level: a well-known fact, but still difficult to characterize and more importantly to mitigate. Current studies are being carried by several groups on developing efficient methods to generate RSSI fingerprint databases. In this work, we should focus on fusing the fingerprint of the beacons by varying the power settings as a means to mitigate the fast fading impairment. We then evaluate the performance of two supervised learning algorithms as a basis to develop an indoor localization mechanism.

### 3.4. Supervised Learning Algorithms

As already stated, the statistics of the Bluetooth signal, mean and standard deviation, show the need of exploring alternative data processing mechanisms towards the development of an RSSI-based localization solution. We base our proposal on the use of the two following classification algorithms [22]:

- **k-NN**: Given a test instance, this algorithm selects the k nearest neighbours, based on a pre-defined distance metric of the training set. In our case, we use the Euclidean distance since our predictor variables (features) share the same type, i.e., the RSSI values, properly fitting the indoor localization problem [22]. Although k-NN uses the most common neighbour of the k located categories (that is the mode of the category) to classify a given test instance, some variations are used (e.g., weighted distances) to avoid removing relevant information. In this paper, we have set the hyperparameter to k = 5 as the best solution, based on some of our preliminary numerical analysis. We use both mentioned versions of the algorithm: the weighted distance (WD) and mode (MD).
- **SVM**: Given the training data, a hyperplane is defined to optimally discriminate between different categories. If linear classifier are used, SVM constructs a line that performs an optimal discrimination. For the non-linear classifier, kernel functions are used, which maximize the margin between categories. In this paper, we have explored the use of linear classifier and Polynomial kernel with two different grades, namely, 2 and 3. Finally, we present only the best results, which were obtained with a Polynomial kernel with a quadratic function [22].

In order to properly justify which of the two mobile devices best fit our needs, we evaluate the accuracy of our proposal using the two classification algorithms. Both devices, BLE4.0 antenna and smartphone, were tested using k-NN and SVM, where k-NN was proven to be the most optimal and efficient algorithm for these types of problems because it works well in a low-dimensional space (in this case, five features) avoiding the curse of dimensionality (the more volume of input, the more training time since it increases at an exponential rate). Although SVM gives a similar precision to k-NN, its runtime is higher because with a view to having a well separated hyperplane, the input space should be high enough [11,23]. We used the data collected during the previously described experimental campaign. For each trial, the data training set consisted of two-thirds and a validation set of one-third of the vectors, randomly selected for each experiment. The results show the mean error of the algorithm executed 50 times.

Table 1 shows that the use of the device equipped with the BLE4.0 antenna provides much better results. A greater accuracy is reported by the BLE4.0 antenna device than for the smartphone. In fact, the results show that the accuracy is almost three times better than the one reported by the smartphone. Based on these results, we decided to use the BLE4-0 antenna device as our experimental tool.

## 4. On the Adequacy of the Bluetooth-Based Localization Platform

This section is devoted to analyse the adequacy of our experimental platform. To do that, first, we performed a preliminary analysis to assess the relevance of each of the five BLE4.0 beacons with respect to a classification model. This analysis has been done using the RSSI reported using different transmission power levels. Furthermore, this study should set the basis for exploring an asymmetric setting of the transmission power. In other words, it is worth exploring if the precision of the localization mechanisms may be improved using different transmission power levels. Obviously, the resulting configuration should be derived from the signal fingerprint of each BLE4.0 beacon.

### 4.1. Relevance of BLE4.0 Beacons

We propose the use of feature selection techniques in order to assess the relevance of each BLE4.0 beacon in the classification model [24]. Although these techniques are used mainly to improve a model, they are also used to identify the importance of the features with the response variable [25]. Here, we use two well-known techniques: the ExtraTrees [26] and Gradient Boosting Classifier [27]. Our choice is based on the fact that both algorithms are robust and accurate. In addition, differently to the Principal Component Analysis and SelectKBest algorithms [28], they do not require any previous parameter tuning. In the following, a brief description of these two algorithms is presented:

- **ExtraTrees** stands for Extremely Randomized Trees, which is an ensemble method that builds multiple models (random trees) for each sample of the training data. Then, all of the predictions are averaged. Default sklearn python library hyperparameters were used.
- **Gradient Boosting Algorithm** is also an ensemble method using decision trees as base models and weighted voting selection method. Furthermore, it makes a prior model every time it is executed. Default sklearn python library hyperparameters were used.

Both algorithms compute a score associated to each feature, which represents the relevance, in percentage, of this feature to the classification process [29].

Table 2 shows the number of samples per BLE4.0 beacon captured at different transmission power levels using the BLE4.0 antenna device. Although the BLE4.0 beacons may operate at eight different transmission power levels, we have not made use of the two lowest levels, namely, Tx=0x07 and Tx=0x08, since they have not been detected over the whole experimental area.

**Figure 8:**
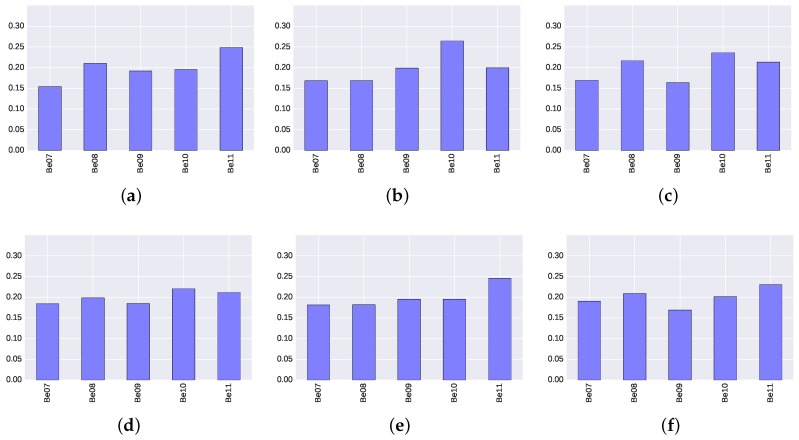
Relevance score of each BLE4.0 beacon for ExtraTrees algorithm for different transmission power (Tx) levels. (**a**) Tx=0x01; (**b**) Tx=0x02; (**c**) Tx=0x03; (**d**) Tx=0x04; (**e**) Tx=0x05; (**f**) Tx=0x06.

**Figure 9:**
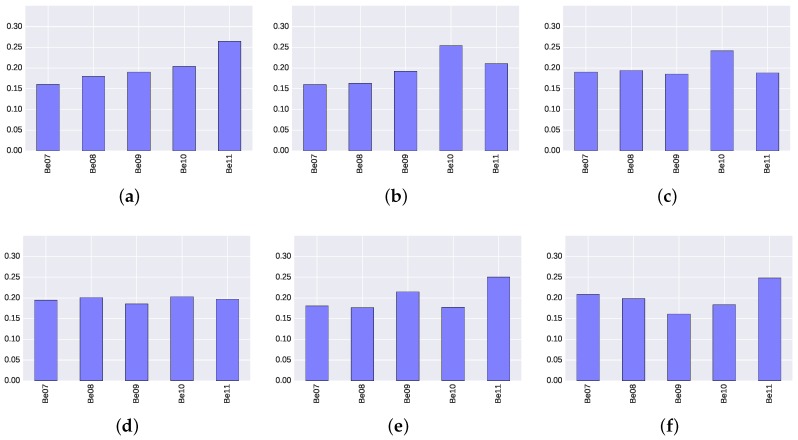
Relevance score of each BLE4.0 beacon for Gradient Boosting Classifier algorithm for different transmission power (Tx) levels. (**a**) Tx=0x01; (**b**) Tx=0x02; (**c**) Tx=0x03; (**d**) Tx=0x04; (**e**) Tx=0x05; (**f**) Tx=0x06.

The ideal situation would be when all BLE4.0 beacons have the same relevance to the classification model, or similarly to find a uniform distribution in the relevance scores. Figure 8 and Figure 9 show the scores for the five BLE4.0 beacons over the six different transmission power levels under study. An analysis of the results clearly show that the transmission power plays a significant role. For instance, Figure 8a shows that, when Tx=0x01, the BLE4.0 beacon ’Be011’ is more relevant to the classification model than all of the other BLE4.0 beacons. However, in the case when the when Tx=0x02, the BLE4.0 beacon ’Be010’ becomes more relevant. Moreover, Figure 8d and Figure 9d, with Tx=0x04, exhibit a more uniform distribution, and all BLE4.0 beacons have a similar relevance in the classification model.

From these results, it is clear that all BLE4.0 beacons exhibit similar relevance scores. They do not deviate more than 5% from the others and none of them exceeds a 30% of the total relevance. These figures allow us to confirm that the experimental set-up is balanced and therefore suitable for exploring the performance of our proposed indoor localization mechanism.

### 4.2. Baseline Evaluation

In this section, we evaluate the accuracy of the two classification algorithms for each one of the six different transmission power levels, i.e., all BLE4.0 beacons operate at the same transmission power level. Table 3 shows that the best accuracy for the k-NN and the SVM algorithms are 65% for Tx=0x03 and 61.7% for Tx=0x06, respectively.

**Figure 10:**
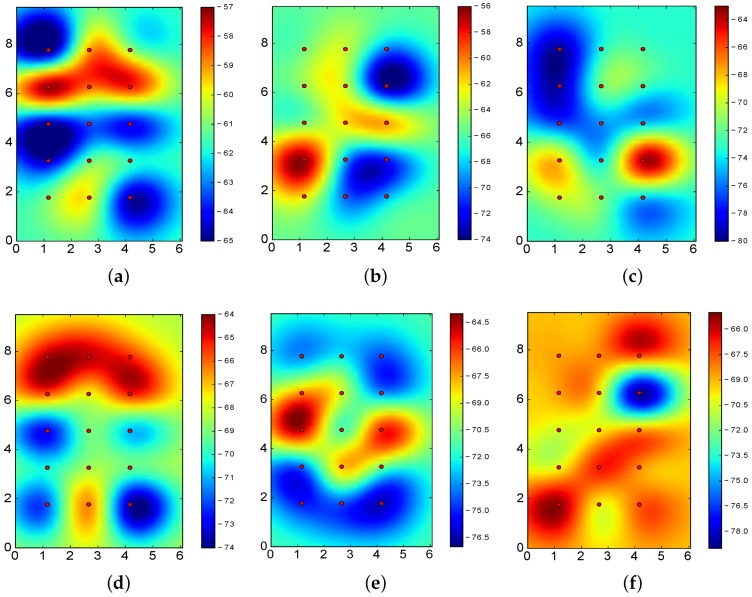
RSSI values for the best (**top**) and worst (**bottom**) transmission power (Tx) level for BLE4.0 beacons ’Be07’, ’Be09’ and ’Be10’ throughout the area captured by the BLE4.0 antenna. (**a**) Be07 with Tx=0x03; (**b**) Be09 with Tx=0x03; (**c**) Be10 with Tx=0x03; (**d**) Be07 with Tx=0x05; (**e**) Be09 with Tx=0x05; (**f**) Be10 with Tx=0x05.

Figure 10 shows the RSSI values for BLE4.0 beacons ’Be07’, ’Be09’ and ’Be11’ when operating at Tx=0x03 and Tx=0x05, i.e., the transmission power levels reporting the best and worst results for the k-NN algorithm.

From the figures, it is clear that better results are obtained when the RSSI reported for the various sectors are clearly differentiated. In particular, Figure 10a–c allows us to properly identify the actual location of the BLE4.0 beacons: the highest RSSI value of the footprint is closely located to the BLE4.0 beacon. However, Figure 10d–f does not exhibit this feature: some of the highest RSSI values are reported far away from the actual BLE4.0 beacon physical location. More specifically, in all these latter cases, the highest RSSI values are reported at two different points. For instance, in the case of BLE4.0 beacon ’Be10’ operating at Tx=0x05 (see Figure 10f), the highest RSSI values are reported at two opposite corners of the experimental area. This signal impairment, known as deep multipath fading, is one of the main obstacles towards the development of robust and accurate BLE-based location mechanisms [7]. In the presence of multipath fading, the information to be derived from the RSSI values of each individual BLE4.0 beacons will definitely mislead the classification process.

Among the various proposals reported in the literature, transmission power control is theoretically one of the most effective approaches for mitigating the multipath effect [30]. However, this process is not as straightforward as it seems. For instance, the results for the BLE4.0 beacon ’Be10’, show that the use of Tx=0x02 may provide some of the best results (see Figure 8b and Figure 9b). However, setting the transmission powers of the BLE4.0 beacons to Tx=0x02 results on the second lowest ranked power transmission configuration (see Table 3). This clearly shows that the setting of the other BLE4.0 beacons play a major role on the overall outcome.

From the previous analysis, it is worth exploring if an asymmetric transmission power setting has a positive impact on the classification. As seen from Figure 10, the different settings of the transmission power of the BLE4.0 beacons may provide lower or higher relevance to the classification process. In the next section, we undertake an in-depth study on this issue.

## 5. Asymmetric Transmission Power

In this section, we start by motivating the study of an asymmetric transmission power setting of the BLE4.0 beacons over the accuracy of the classification model. We then find the setting by examining all of the transmission power setting/BLE4.0-beacon combinations. Our results are reported in terms of the local and global accuracy. The former provides the accuracy of the classification model per each one of the 15 sectors, while the latter refers to the accuracy over the whole experimental area.

### 5.1. Fingerprint as a Function of the Transmission Power

In the previous section, we have found that the accuracy of the classification process heavily depends on the transmission power of the BLE4.0 beacons. More specifically, we noticed that, in the presence of the multipath fading impairment, the classification process is heavily penalized. It is therefore worth exploring an asymmetric transmission power setting of the BLE4.0 beacons. Such a setting should allow us to exploit the characteristics of the fingerprint as a means to improve the accuracy of the identification process.

**Figure 11:**
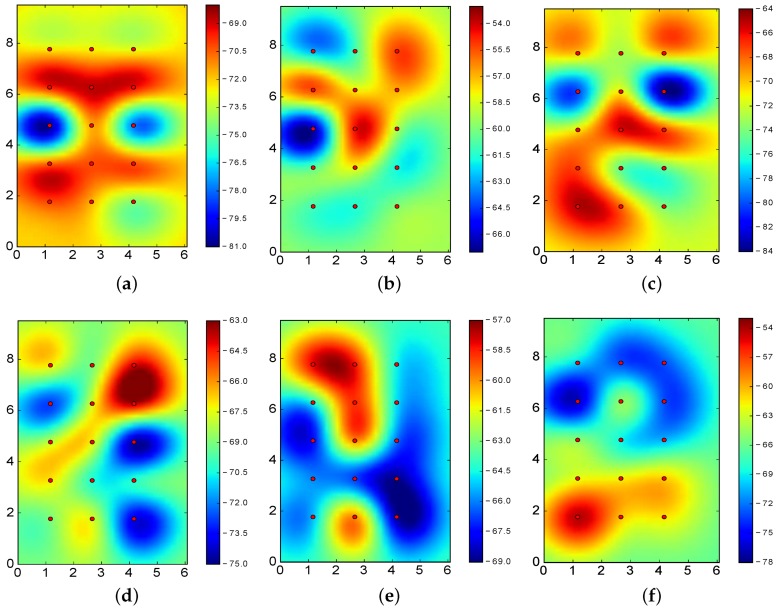
RSSI values for different transmission power levels (*Tx*) for BLE4.0 beacons ’Be11’, ’Be07’ and ’Be08’. (**a**) ’Be11’ with Tx=0x03; (**b**) ’Be07’ with Tx=0x01; (**c**) ’Be08’ with Tx=0x05; (**d**) ’Be11’ with Tx=0x04; (**e**) ’Be07’ with Tx=0x04; (**f**) ’Be08’ with Tx=0x01.

In order to further motivate our work, we start by visually examining the RSSI values associated to the fingerprint of three of the five BLE4.0 beacons used in our testbed, namely, BLE4.0 beacons ’Be11’, ’Be07’ and ’Be08’ (see Figure 11). Figure 11a,d show the RSSI values for BLE4.0 beacon ’Be11’ when operating at two different transmission power levels. The values shown in Figure 11d exhibits better characteristics: the highest RSSI value is closely located and delimited around the area where the BLE4.0 beacon ’Be11’ is placed, i.e., the upper right corner of the figure. On the contrary, the values shown in Figure 11a does not allow us to easily identify the location of the BLE4.0 beacon ’Be11’. The results for the other two BLE4.0 beacons exhibit similar characteristics. We further notice that the most useful fingerprints for BLE4.0 beacon ’Be07’ and ’Be11’ share the same transmission power level Tx=0x04. However, in the case of BLE4.0 beacon ’Be08’, the transmission power setting that provides better results is Tx=0x01. Therefore, it is worth exploring the setting of the transmission power setting as a way to improve the accuracy of the identification algorithms.

### 5.2. On Deriving the Best Asymmetric Transmission Power Setting

In this section, we conduct an ad hoc process to find the best transmission power setting by evaluating all the transmission power setting/BLE4.0-beacon combinations. Each combination is evaluated in terms of its local and global accuracy. In our case, our platform consists of five BLE4.0 beacons operating at one of six possible transmission power levels. This involves a total of 7776 combinations to be processed.

### Case 1: Asymmetric Transmission Power for k-NN

**Figure 12:**
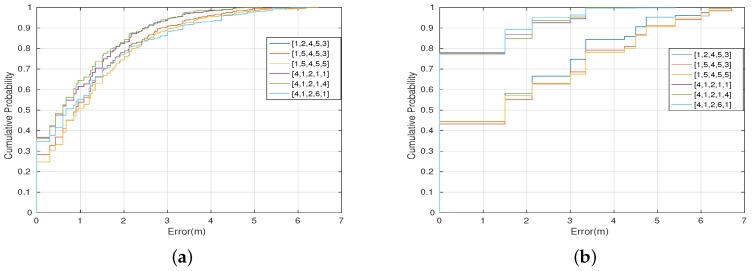
Positioning error for k-NN (with k = 5) using (**a**) weighted distance; (**b**) mode. In both plots, the three best and the three worst combined transmission power for each BLE4.0 beacon are shown.

Figure 12 shows the overall cumulative positioning error for the three best and the three worst combined transmission power levels for k-NN, using both versions of the classification algorithm, namely, weighted distance (a) and mode (b). The most relevant transmission power level combination is the one with the configuration: BLE4.0 beacon ’Be07’ with Tx=0x04, BLE4.0 beacon ’Be08’ with Tx=0x01, BLE4.0 beacon ’Be09’ with Tx=0x02, BLE4.0 beacon ’Be10’ with Tx=0x01 and BLE4.0 beacon ’Be11’ with Tx=0x01, which, in the following, will be represented by $\left[4,1,2,1,1\right]$ for short. This vector contains the transmission power level assigned to BLE4.0 beacons ’Be07’, ’Be08’, ’Be09’, ’Be10’, and ’Be11’, respectively. The figure shows that this setting limits the positioning error to less than 3 m in 95% of the times, for both versions of the k-NN classification algorithm. For the worst configurations, the 95% of the cumulative error is achieved with errors of 4 m (WD) and 5.5 m (MD), respectively.

**Figure 13:**
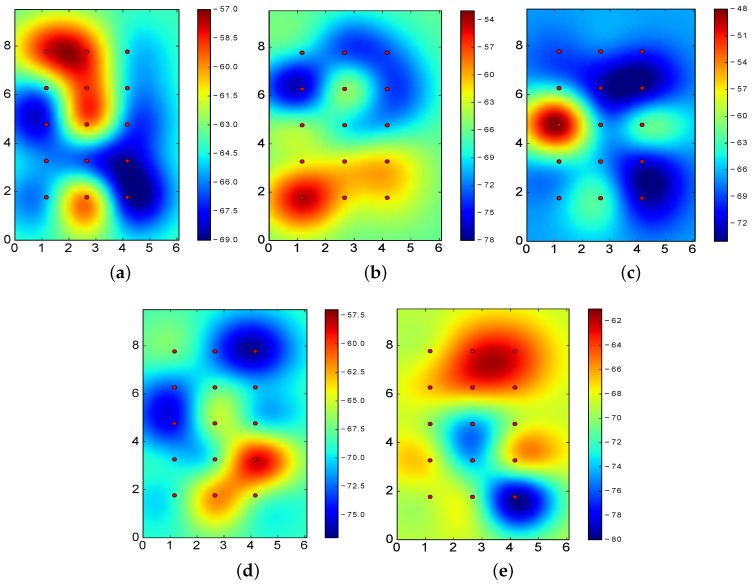
RSSI values using the most relevant transmission power (Tx) level setting for each BLE4.0 beacon: [4,1,2,1,1]. (**a**) ’Be07’ with Tx=0x04; (**b**) ’Be08’ with Tx=0x01; (**c**) ’Be09’ with Tx=0x02; (**d**) ’Be10’ with Tx=0x01; (**e**) ’Be11’ with Tx=0x01.

Figure 13 shows the RSSI values for the most relevant transmission power levels. The results show that the location of each BLE4.0 beacon is properly identified by the RSSI fingerprint. That is, such sectors are quite relevant to the classification algorithms.

Table 4 shows the local accuracy for each sector (15 in total) using the most relevant transmission power levels. The results show that the best accuracy is reported for the sectors close to the BLE4.0 beacons, while the accuracy deteriorates as a function of the distance.

Comparing the results in Table 4 with those in Figure 13, we notice that the midpoint sector, with an accuracy of 18.10%, does not have a distinctive RSSI differentiated from the others, i.e., the RSSI values of all the BLE4.0 beacons are very constant in this sector.

In the case of BLE4.0 beacon ’Be09’, Figure 13c, we have a representative RSSI totally different from the one reported for the other sectors. This guarantees a good classification at this sector with a 100% of local accuracy (see Table 4). Moreover, from Figure 4b, we can observe that sector 7 (the closest to BLE4.0 beacon ’Be09’) has a characteristic RSSI totally different from the others. This result confirms the benefits of counting with a sector with a distinctive RSSI fingerprint: a substantial improvement, locally and globally, on the positioning accuracy.

### Case 2: Asymmetric Transmission Power for SVM

Similarly to the previous section, we carried out an analysis using the SVM algorithm. In this case, we found that the most relevant transmission power levels were exactly the same as for the k-NN algorithm: [4,1,2,1,1]. The global accuracy was 75.57% and the RSSI propagation heatmap is also shown in Figure 13.

**Figure 14:**
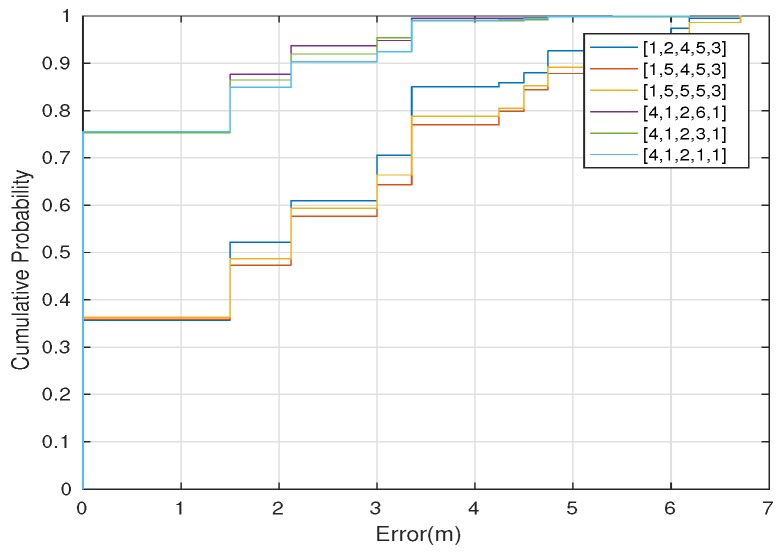
Positioning error for SVM (with a quadratic polynomial kernel function). In both plots, the three best and the three worst combined transmission power for each BLE4.0 beacon are shown.

Figure 14 shows the positioning error for the three best and worst combined transmission power levels for SVM, which are very similar to the ones obtained with k-NN. The figure shows that, for the three best transmission power level settings, the positioning error is lower than 3 m in 95% of the times. For the three worst configurations, a positioning error of less than 6 meters is obtained with a cumulative probability of 0.95.

Table 5 shows the local accuracy for each sector (15 in total) using the most relevant transmission power level setting ([4,1,2,1,1]), showing a very similar behaviour as k-NN. We can observe that the areas that have a weak characterization by RSSI propagation will have a worst local accuracy, as observed for the midpoint with only a 19.83% of local accuracy.

Our results confirm that a proper setting of the transmission power of each BLE4.0 beacon has a positive impact on the performance of both classification algorithms SVM and k-NN by a proper setting, we mean to make use of the RSSI map of each BLE4.0 beacon allowing us to differentiate one sector from another.

Although we do not have conclusive evidence on the nature and extend of the impact of the architecture of our lab premises over the signal, we notice that the highest power levels have been assigned to BLE4.0 beacons ’Be08’, ’Be10’ and ’Be11’, the ones closer to the window and the open corridor, while lower transmission power levels have been assigned to BLE4.0 beacons ’Be07’ and ’Be09’, the ones located at the plasterboard wall. As mentioned in the introduction, recent studies have shown that the use of a priori floor plan information may enable the development of more accurate wireless indoor localization schemes [12].

### 5.3. Asymmetric Transmission Power Setting

Table 6 and Table 7 show the results for different transmission power settings obtained for both classification algorithms: k-NN and SVM. For each algorithm, two different transmission power settings were used: best configuration using symmetric transmission power setting ([3,3,3,3,3] for k-NN and [6,6,6,6,6] for SVM; and best configuration using asymmetric transmission power level setting ([4,1,2,1,1] for both k-NN and SVM). From the results in Table 6, it is clear that properly setting the transmission power of each BLE4.0 beacon, the cumulative positioning error can be substantially reduced. Furthermore, k-NN (MD) reports in general slightly better results than k-NN (WD) and SVM. These results are corroborated with the ones presented in Table 7. The results show that k-NN (MD) with the asymmetric transmission power setting exhibits a lower mean error, approximately 0.07 m lower than the obtained by SVM.

Finally, Table 8 shows the global accuracy using different asymmetric transmission power level settings (the five worst and the five best results), and using all symmetric transmission power settings. We can observe that, for SVM, the worst and best asymmetric transmission power settings report an accuracy rate of 35.70% and 75.57%, respectively: the latter being substantially better to the 61.70% reported using the best results using a symmetric transmission power setting, i.e., [6-6-6-6-6]. From the results shown in the table, we notice that the k-NN algorithm reports higher scores in all transmission power settings—for both, the five worst and five best settings than those reported when the SVM algorithm is applied. We further notice that both algorithms rank the same transmission power setting, namely, [4-1-2-1-1] as the best one.

A further analysis of the results depicted in Table 8 show that both algorithms clearly classify the transmission power of some of the BLE4.0 beacons as the best choices. This is the case, for instance, for BLE4.0 beacons ’Be08’ whose best transmission power is Tx=0x01 for all five best settings reported by both algorithms. As for the case of BLE4.0 beacons ’Be07’ and ’Be09’, the most recommended values are Tx=0x04 and Tx=0x02, respectively. As previously discussed for the case of BLE4.0 beacon ’Be09’ (see Figure 13c), the classification process greatly benefits when the RSSI provides the means to identify the location of the reference BLE4.0 beacon. Our results seem to confirm the benefits of using the transmission power setting whose RSSI better contribute to the classification process. However, in the case of the SVM algorithm, we notice that the transmission power value used by BLE4.0 beacon ’Be09’ in the fourth best ranked setting is the same as the one used in the worst ranked setting. We should further explore the relevance of the individual transmission power level as a major source of information and more importantly, the impact of the asymmetric power levels setting as a means to overcome the multipath fading impairment.

### 5.4. On the Relevance of the Individual RSSI Values

With the purpose of evaluating the relevance of the information provided by the RSSI values as a major source of information to guide the classification process, we look at the ranking of the individual transmission power values used by each one of the BLE4.0 beacons. In the previous section, we noticed that in the worst and fourth best transmission power settings reported by the SVM results, the transmission power of BLE4.0 beacon ’Be09’ has been set to Tx=0x04. In order to explore further this issue, we looked for each one of the BLE4.0 beacons, and the worst ranked setting making use of the transmission power values for each of the BLE4.0 beacons. We carry out this study only for the k-NN algorithm use mode. Similar conclusions may be derived from an analysis of the results reported by SVM. In fact, the aforementioned case for BLE4.0 beacon ’Be09’ provided us the basis of our analysis.

Table 9 shows the rankings among the worst transmission power settings of the transmission power used in the best setting by each BLE4.0 beacon. As seen from the table, the transmission power used in the best case for all BLE4.0 beacons also make part of a reduced number of the worst settings. For instance, in the case of BLE4.0 beacon ’Be09’, the transmission power value, Tx=0x02, having been visually characterized as an excellent source of information, makes up part of the 0.5% worst settings. These results clearly show that the RSSI derived from the transmission power used by an individual source does not guarantee by itself the best classification process. We should then further explore the use of an asymmetric transmission power setting as a means to mitigate the multipath fading impairment. This analysis should provide us a basis to identify the approach to be used to improve the classification process.

### 5.5. On Mitigating the Multipath Fading Impairment

In this section, we start by taking a closer look at the transmission power setting [1-1-1-1-1]. Our choice is based on the fact that both classification algorithms ranked this setting as the fourth best symmetric setting (see Table 8). Furthermore, we notice that, in the best setting, the transmission power of three out of the five beacons has been set to Tx=0x01. Our main aim is therefore to provide a further insight on the improvement on the quality of the information provided to the classification algorithms.

From Figure 11b,e, we can clearly identify the presence of the multipath fading effect. From the figures, one may think that changing the transmission power of BLE4.0 beacon ’Be07’ to Tx=0x04 will lead to similar or even worse results than the ones reported for Tx=0x01. However, our results show that by simply changing the setting of BLE4.0 beacon ’Be07’, i.e., using the setting [4-1-1-1-1], the global accuracy reported by the k-NN algorithm considerably improves from 62.10 to 69.9%. This can be explained by a close look at the results reported in Figure 13 for BLE4.0 beacons ’Be07’ and ’Be08’. From the figures, it is clear that by setting the transmission power of BLE4.0 beacon ’Be07’ to Tx=0x04 and ’Be08’ to Tx=0x04, the highest RSSI levels of BLE4.0 beacon ’Be08’ located at the bottom part of the figure help to mitigate the effect of the multipath fading impairment.

Let us now consider the transmission power setting [4-4-4-4-4]. As shown in Table 8, both classification algorithms have ranked this setting as the second best one among the symmetric transmission power settings. Our results reported that by simply changing the power setting to [4-4-2-4-4], the global accuracy of increases from 64.7% (see Table 8) to 69.2%, i.e., an improvement of almost 5%. However, if we set the transmission power to [1-4-4-4-3], the global accuracy drops to 62.2%, i.e., a decrease close to 2.5%. In fact, we could expect a higher drop since the RSSI values for BLE4.0 beacon ’Be07’ (see Figure 11a) does not allow us to clearly identify the actual location of BLE4.0 beacon ’Be11’. Let us now consider the setting [1-4-4-4-4]. From our previous analysis and the RSSI values of BLE4.0 beacon ’Be07’ when using Tx=0x01 (see Figure 11b), we may not expect a higher drop than the one reported for the previously analysed [4-4-4-4-3] setting. However, our results report a global accuracy of 57.5% for this latter setting. That is to say, the accuracy drops more than 7% with respect to the symmetric setting [4-4-4-4-4].

The above analysis sets the basis towards deriving a methodology allowing us to enhance the performance of the classification algorithms. From the results reported in Table 8, we may start by setting the transmission power of all the BLE4.0 beacons to the same values; all symmetric settings rank around the median. The use of a database of RSSI values of all of the BLE4.0 beacons at different transmission power levels may be used to derive a setting offering better results. In fact, various works recently reported in the literature are working on the creation of such databases [31]. Since finding the best setting depends on the combination and features of the RSSI maps, one of the first approaches is to study different combinatorial optimization algorithms, e.g., genetic algorithms. In other words, one may start by setting a symmetric transmission power setting, and, based on the RSSI levels reported using different transmission power settings, the quality of the information to be provided to the classification algorithms may be enhanced.

From this analysis, we can conclude that:

- Although it is important to classify the sectors with a distinctive RSSI, the percentage of settings obtained is not a considerable matching of the combinations between the two classification algorithms.
- The RSSI value of a given BLE4.0 beacon proves to be a useful, but not definitely, the main source of information on setting the best transmission power setting.
- An asymmetric transmission power setting may prove useful on mitigating the information to be provided to the classification algorithms due to the multipath fading effect.

## 6. Conclusions

This study has revealed some useful insight on the required tool characteristics to calibrate an accurate BLE4.0-assisted indoor localization mechanism. Based on the constraints imposed by the smartphones, mainly the limited sampling rate and antennas, the basic requirements of the calibration platform can be simply stated as: (i) the use of a hardware transmitter with different transmission power levels; (ii) the use of BLE4.0 antenna; and (iii) an evaluation of the relevance of the RSSI of each BLE4.0 beacon to the classification models taking into account their placement and transmission powers.

Although we have not been able to fully explain the extent and nature of the impact of the architectural features over the RSSI metric, we have paid attention to describing the lab floor. Our results provide some insight on the relevance of knowing the placement of the BLE4.0 beacons with respect to reflective surfaces, e.g., windows and plasterboard walls.

In this work, we have presented the importance of using a good BLE4.0 receiver—in this case, a BLE4.0 antenna, for indoor localization, improving the accuracy significantly over the one obtained using a smartphone.

Our approach integrates the study of a balanced Bluetooth sensor topology analysing the relevance of this BLE4.0 beacons for the classification algorithms, Gradient Boosting Classifier and Extra Trees being a robust and accurate solution.

Our immediate research efforts will be focused on improving the experimental set-up to further evaluate the use of different transmission power levels using the classification algorithms. Our main goal is to develop a methodology allowing us to find the optimal setting of the transmission power levels and placement of the BLE4.0 beacons. We believe that these two parameters should greatly improve the local and global accuracy of our proposal.

Moreover, we also have in mind to extend this research to incorporate different study of the Bluetooth network topology, trying to improve the local and global accuracy. The use of other Machine Learning algorithms is quite important to improve the accuracy and, of course, the different filters to identify the outliers.

Another major task in our immediate research plans is to study different combinatorial optimization algorithms (e.g., genetic algorithms) to perform the asymmetric assignment optimally and automatically.

## Figures and Tables

**Table 1:** Global accuracy for k-NN using mode (with k = 5) and SVM (with a quadratic polynomial kernel function) algorithms for transmission power Tx=0x04. Best results are shown in bold.

Algorithm	Smartphone	BLE4.0 Antenna
k-NN	21%	64.6%
SVM	22.4%	60.6%

**Table 2:** Sample sizes of the RSSI captured using the BLE4.0 at various transmission power (Tx) levels.

Transmission Power	Sample Size per BLE4.0 Beacon
Tx=0x01	5003
Tx=0x02	5246
Tx=0x03	4844
Tx=0x04	5134
Tx=0x05	4697
Tx=0x06	4198

**Table 3:** Global accuracy using BLE4.0 antenna for k-NN (with *k* = 5) using mode and SVM (with a quadratic polynomial kernel function) algorithms for different transmission power (Tx) levels. Best results are shown in bold.

Transmission Power	Algorithm	
	k-NN	SVM
Tx=0x01	62.3%	57.7%
Tx=0x02	61.5%	52.6%
Tx=0x03	**65.0%**	58.0%
Tx=0x04	64.6%	60.6%
Tx=0x05	56.6%	50.4%
Tx=0x06	63.8%	**61.7%**

**Table 4:** Local accuracy in each sector of our experimental area with the most relevant transmission power level for k-NN using mode (with k = 5). The centre shows the accuracy (in %) of each sector. Corners and middle-left hand are the position of BLE4.0 beacons with BeXY name. The most relevant transmission power level was [4,1,2,1,1].

**Be07**				**Be11**
	81.31	71.43	84.62	
	30.10	70.69	84.11	
**Be09**	100.00	18.10	52.88	
	28.95	71.17	53.19	
	72.10	86.92	77.59	
**Be08**				**Be10**

**Table 5:** Local accuracy in each sector of our experimental area with the most relevant transmission power level for SVM (with a quadratic polynomial kernel function). The centre shows the accuracy (in %) of each sector. Corners and middle-left hand are the position of BLE4.0 beacons with BeXY name. The most relevant transmission power level was [4,1,2,1,1].

**Be07**				**Be11**
	85.00	80.70	99.07	
	11.50	69.17	76.64	
**Be09**	70.43	19.83	51.97	
	20.18	68.38	27.66	
	52.33	87.85	91.38	
**Be08**				**Be10**

**Table 6:** Cumulative positioning error with different transmission power (Tx) level settings for k-NN (with k = 5) using weighted distance (WD) and mode (MD); and SVM (with a quadratic polynomial kernel function). Best results are shown in bold.

Algorithm - Tx Setting	Cumulative Positioning Error				
	0 m	≤1 m	≤2 m	≤3 m	≤4 m
k-NN (WD) - [3,3,3,3,3]	33.27%	57.22%	77.53%	88.15%	95.12%
k-NN (WD) - [4,1,2,1,1]	36.15%	64.47%	82.92%	94.03%	98.70%
k-NN (MD) - [3,3,3,3,3]	65.00%	65.00%	74.57%	81.36%	89.26%
**k-NN (MD) - [4,1,2,1,1]**	**77.89%**	**77.89%**	**86.74%**	**92.52%**	**99.68%**
SVM - [6,6,6,6,6]	61.70%	61.70%	72.81%	77.22%	88.40%
SVM - [4,1,2,1,1]	75.57%	75.57%	84.92%	90.33%	99.12%

**Table 7:** Mean error for k-NN (with k = 5) using weighted distance (WD) and mode (MD); and SVM (with a quadratic polynomial kernel function) with the same and the most relevant transmission power level (Tx). Best results are shown in bold.

Algorithm - Tx Setting	Mean Error (m)
k-NN (WD) - [3,3,3,3,3]	1.16
k-NN (WD) - [4,1,2,1,1]	0.57
k-NN (MD) - [3,3,3,3,3]	1.11
**k-NN (MD) - [4,1,2,1,1]**	**0.51**
SVM - [6,6,6,6,6]	1.17
SVM - [4,1,2,1,1]	0.58

**Table 8:** Accuracy results for the k-NN using mode (with k = 5) (right) and SVM localization (with a quadratic polynomial kernel function) (left) algorithms. Worst and best settings using different asymmetric transmission power settings, and the best symmetric transmission power level settings (shown in *italic* font). Best results are shown in bold.

SVM		k-NN	
Tx Setting	Accuracy	Tx Setting	Accuracy
[1-2-4-5-3]	35.70%	[1-5-4-5-3]	43.23%
[1-5-4-5-3]	35.91%	[1-2-4-5-3]	44.48%
[1-5-5-5-3]	36.28%	[1-5-4-5-5]	44.53%
[1-5-5-2-3]	36.69%	[1-3-4-5-3]	44.54%
[1-2-4-2-3]	36.73%	[1-3-4-2-3]	44.58%
*[2-2-2-2-2]*	*52.68%*	*[5-5-5-5-5]*	*56.70%*
*[5-5-5-5-5]*	*50.41%*	*[2-2-2-2-2]*	*61.50%*
*[1-1-1-1-1]*	*57.74%*	*[1-1-1-1-1]*	*62.10%*
*[3-3-3-3-3]*	*57.90%*	*[6-6-6-6-6]*	*63.80%*
*[4-4-4-4-4]*	*60.70%*	*[4-4-4-4-4]*	*64.70%*
*[6-6-6-6-6]*	*61.70%*	*[3-3-3-3-3]*	*65.00%*
[4-1-2-3-2]	73.86%	[3-1-2-1-1]	75.96%
[4-1-4-1-1]	74.29%	[4-1-2-3-4]	76.87%
[4-1-2-6-1]	75.36%	[4-1-2-6-1]	77.23%
[4-1-2-3-1]	75.36%	[4-1-2-1-4]	77.45%
[4-1-2-1-1]	75.57%	**[4-1-2-1-1]**	**77.89%**

**Table 9:** Ranking of the transmission power values used by each BLE4.0 beacon for k-NN using mode (with k = 5) results.

BLE4.0 Beacon	Ranking
Be07	3.7%
Be08	0.9%
Be09	0.5%
Be10	5.0%
Be11	2.5%

## Abbreviations

The following abbreviations are used in this manuscript:

RSSI	Received Signal Strength Indication
BLE4.0	Bluetooth Low Energy 4.0
k-NN	k-Nearest Neighbour
SVM	Support Vector Machine
AP	Access Point
Tx	Transmission Power
dB	Decibel
dBm	Decibel-milliwatts
MD	Mode
WD	Weighted Distance

## References

[1] Shuo S., Hao S., Yang S. Design of an experimental indoor position system based on RSSI. Proceedings of the 2nd International Conference on Information Science and Engineering.

[2] Feldmann S., Kyamakya K., Zapater A., Lue Z. An indoor bluetooth-based positioning system: Concept, implementation and experimental evaluation. Proceedings of the International Conference on Wireless Networks.

[3] Shukri S., Kamarudin L., Cheik G.C., Gunasagaran R., Zakaria A., Kamarudin K., Zakaria S.S., Harun A., Azemi S. Analysis of RSSI-based DFL for human detection in indoor environment using IRIS mote. Proceedings of the 3rd IEEE International Conference on Electronic Design (ICED).

[4] Rappaport T. (2001). Wireless Communications: Principles and Practice.

[5] Martínez-Gómez J., del Horno M.M., Castillo-Cara M., Luján V.M.B., Barbosa L.O., García-Varea I. (2016). Spatial statistical analysis for the design of indoor particle-filter-based localization mechanisms. Int. J. Distrib. Sens. Netw..

[6] Onishi K. Indoor position detection using BLE signals based on voronoi diagram. Proceedings of the International Conference on Intelligent Software Methodologies, Tools, and Techniques.

[7] Palumbo F., Barsocchi P., Chessa S., Augusto J.C. A stigmergic approach to indoor localization using bluetooth low energy beacons. Proceedings of the 12th IEEE International Conference on Advanced Video and Signal Based Surveillance.

[8] Wang Q., Feng Y., Zhang X., Su Y., Lu X. (2016). IWKNN: An effective bluetooth positioning method based on isomap and WKNN. Mob. Inf. Syst..

[9] Faragher R., Harle R. An analysis of the accuracy of bluetooth low energy for indoor positioning applications. Proceedings of the 27th International Technical Meeting of The Satellite Division of the Institute of Navigation.

[10] Peng Y., Fan W., Dong X., Zhang X. An Iterative Weighted KNN (IW-KNN) Based Indoor Localization Method in Bluetooth Low Energy (BLE) Environment. Proceedings of the 2016 International IEEE ConferencesUbiquitous Intelligence & Computing, Advanced and Trusted Computing, Scalable Computing and Communications, Cloud and Big Data Computing, Internet of People, and Smart World Congress.

[11] Zhang L., Liu X., Song J., Gurrin C., Zhu Z. A comprehensive study of bluetooth fingerprinting-based algorithms for localization. Proceedings of the 27th IEEE International Conference on Advanced Information Networking and Applications Workshops (WAINA).

[12] Leitinger E., Meissner P., Rüdisser C., Dumphart G., Witrisal K. (2015). Evaluation of position-related information in multipath components for indoor positioning. IEEE J. Sel. Areas Commun..

[13] Wang Q., Guo Y., Yang L., Tian M., Pan Z., Cheok A.D., Müller W., Zhang M. (2017). An indoor positioning system based on ibeacon. Transactions on Edutainment XIII.

[14] Kriz P., Maly F., Kozel T. (2016). Improving indoor localization using bluetooth low energy beacons. Mob. Inf. Syst..

[15] Faragher R., Harle R. (2015). Location fingerprinting with bluetooth low energy beacons. IEEE J. Sel. Areas Commun..

[16] Paek J., Ko J., Shin H. (2016). A measurement study of ble ibeacon and geometric adjustment scheme for indoor location-based mobile applications. Mob. Inf. Syst..

[17] Perera C., Aghaee S., Faragher R., Harle R., Blackwell A. (2017). A contextual investigation of location in the home using bluetooth low energy beacons. arXiv.

[18] Pei L., Zhang M., Zou D., Chen R., Chen Y. (2016). A survey of crowd sensing opportunistic signals for indoor localization. Mob. Inf. Syst..

[19] Jaalee Beacon IB0004-N Plus. https://www.jaalee.com/.

[20] Anagnostopoulos G.G., Deriaz M., Konstantas D. Online self-calibration of the propagation model for indoor positioning ranging methods. Proceedings of the International Conference on Indoor Positioning and Indoor Navigation (IPIN).

[21] Trendnet Micro Bluetooth USB Adapter. https://www.trendnet.com/products/USB-adapters/TBW-107UB/.

[22] Brownlee J. (2016). Spot-check classification algorithms. Machine Learning Mastery with Python.

[23] Breiman L. (2001). Statistical modeling: The two cultures (with comments and a rejoinder by the author). Stat. Sci..

[24] Brownlee J. (2016). Feature selection. Machine Learning Mastery with Python.

[25] Rivas T., Paz M., Martín J., Matías J.M., García J., Taboada J. (2011). Explaining and predicting workplace accidents using data-mining techniques. Reliab. Eng. Syst. Saf..

[26] Geurts P., Ernst D., Wehenkel L. (2006). Extremely randomized trees. Mach. Learn..

[27] Brownlee J. (2016). Ensemble methods. Machine Learning Mastery with Python.

[28] Pedregosa F., Varoquaux G., Gramfort A., Michel V., Thirion B., Grisel O., Blondel M., Prettenhofer P., Weiss R., Dubourg V. (2011). Scikit-learn: Machine learning in python. J. Mach. Learn. Res..

[29] Li J., Cheng K., Wang S., Morstatter F., Trevino R.P., Tang J., Liu H. (2016). Feature selection: A data perspective. arXiv.

[30] Rahim A., Dimitrova R., Finger A. Techniques for Bluetooth Performance Improvement. https://pdfs.semanticscholar.org/3205/2262d3c152a3cc947acbc7b325debe9cbeef.pdf.

[31] Chen L., Li B., Zhao K., Rizos C., Zheng Z. (2013). An improved algorithm to generate a Wi-Fi fingerprint database for indoor positioning. Sensors.

